# [Expression of Concern] Expression and significance of glucose-regulated protein 78 in human osteosarcoma

**DOI:** 10.3892/ol.2025.15269

**Published:** 2025-09-15

**Authors:** Yongkui Zhang, Nianhu Li, Dongli Wang, Yiqiang Chen, Gang Li

Oncol Lett 9: 2268–2274, 2015; DOI: 10.3892/ol.2015.3030

Following the publication of this paper, it was drawn to the Editor's attention by a concerned reader that, regarding the immunofluorescence staining images shown in Figs. 1 and 4, an area of overlapping data was identifiable, albeit the images were presented at apparently different magnifications, suggesting that the presentation of one of these two figures was erroneous.

The authors were contacted by the Editorial Office to offer an explanation for this apparent anomaly in the presentation of the data in this paper; however, up to this time, no satisfactory response has been received. Owing to the fact that the Editorial Office has been made aware of potential issues surrounding the scientific integrity of this paper, we are issuing an Expression of Concern to notify readers of this potential problem while the Editorial Office continues to investigate this matter further.

